# Abundance, composition and activity of denitrifier communities in metal polluted paddy soils

**DOI:** 10.1038/srep19086

**Published:** 2016-01-07

**Authors:** Yuan Liu, Yongzhuo Liu, Huimin Zhou, Lianqing Li, Jinwei Zheng, Xuhui Zhang, Jufeng Zheng, Genxing Pan

**Affiliations:** 1 Institute of Resource, Ecosystem and Environment of Agriculture, Nanjing Agricultural University, 1 Weigang, Nanjing 210095, China; 2 Department of Bioengineering, College of Life Sciences, Huaibei Normal University, 235000, Huaibei, Anhui Province, China; 3 College of Resource and Environment Sciences, Henan Institute of Science and Technology, Xinxiang City, Henan 453003, China

## Abstract

Denitrification is one of the most important soil microbial processes leading to the production of nitrous oxide (N_2_O). The potential changes with metal pollution in soil microbial community for N_2_O production and reduction are not well addressed. In this study, topsoil samples were collected both from polluted and non-polluted rice paddy fields and denitrifier communities were characterized with molecular fingerprinting procedures. All the retrieved *nirK* sequences could be grouped into neither α- nor β- *proteobacteria*, while most of the *nosZ* sequences were affiliated with α-*proteobacteria*. The abundances of the *nirK* and *nosZ* genes were reduced significantly in the two polluted soils. Thus, metal pollution markedly affected composition of both *nirK* and *nosZ* denitrifiers. While the total denitrifying activity and N_2_O production rate were both reduced under heavy metal pollution of the two sites, the N_2_O reduction rate showed no significant change. These findings suggest that N_2_O production activity could be sensitive to heavy metal pollution, which could potentially lead to a decrease in N_2_O emission in polluted paddies. Therefore, metal pollution could have potential impacts on soil N transformation and thus on N_2_O emission from paddy soils.

Atmospheric concentration of nitrous oxide (N_2_O), the most radiative greenhouse gas for global warming, has been increasing constantly since 1980 and reached 319 10^−9^ mol mol^−1^ in global air by 2005^1^. Agriculture accounts for about 60% of the global anthropogenic N_2_O emission, with rice paddies being a major contributor^2^,^3^,^4^. Total N_2_O emission from China’s rice paddies was estimated at 29.0 Gg N_2_O per year, contributing by 7–11% to the nation’s total greenhouse gas emission from agriculture^4^.

The activity of denitrification, a rate-limiting process of N_2_O production in soil, could be inhibited by elevated soil metal concentration. Lab incubation study showed that metal addition of Cd, Cu or Zn up to 500 mg kg^−1^ significantly decreased denitrification in wetland sediment, being greatest by Cd followed by Zn and Cu^5^. Similarly, metal loading such as Zn up to 200 mg kg^−1^ reduced the activity of denitrifying enzyme in rice soils across a wide range of soil parent materials^6^. Likewise, in pasture soil, soil denitrification activity was negatively affected by heavy metal^7^, in a proportional response to gradient addition of Cu, Cr, and As concentrations over 50–1300 mg kg^−1^. Moreover, potential denitrification activity was observed significantly reduced with Cu concentration up to 1300 mg kg^−1^, whereas gene abundance of *nirK* community was unchanged in metal polluted rice paddies from South China^8^. Different soil microbial communities were involved in multiple steps in the reduction of nitrate to nitrogen (N_2_), and a process-specific microbial community could differ from another in heavy metal tolerance^9^, and thus, any selective inhibition of N_2_O reductase by heavy metals could enhance N_2_O release in soil.

Release of N_2_O from denitrification in soil was largely determined by N_2_O production and the subsequent N_2_O reduction. As a preceding process of N_2_O production, the reduction of nitrite (NO_2_^−^) to nitric oxide (NO) is catalyzed by nitrite reductases (NirK and NirS) mediated by denitrifiers, which had been well known distinguishable from nitrate respiring bacteria^10^. NirK is a member of the multi-copper oxidase metalloprotein family, whereas NirS contains a cytochrome cd_1_ active site. The nitrite reductase encoded by *nirK* or *nirS* gene was related with Cu and Fe, respectively. The N_2_O reduction, the final step in the denitrification, was catalyzed by nitrous oxide reductase (N_2_OR), which was encoded by gene *nosZ* but not present in all denitrifiers enzymes^11^. While both Nir and N_2_OR enzymes were known sensitive to environmental stresses^12^, and the study of changes in denitrifier communities with functional genes (*nirS*/*nirK* and *nosZ*) were intensively reported in rice paddies^13^, upland croplands^14^ and forest soils^15^. The effects of heavy metal on abundance of *nirK* and/or *nosZ* gene in soils had been studied generally in laboratory with spiked metals^16^,^17^,^18^. Such studies, however, were not directed to predicting the long-term effects of soil metal contamination as microbial community could have different adaption to pollutants and soil chemical immobilization reactions in field soils^19^,^20^. Yet, the changes in abundance and composition of denitrifier communities to long term heavy metal pollution in rice paddies are still unclear.

In China, large areas of rice paddies had been under pollution stresses by multiple heavy metals in the Yangtze River delta^21^,^22^, in the Pearl River delta^23^ and in the Jiangxi province^24^. Different pedogenesis and soil properties could mask microbial community changes and biogeochemical functioning in long-term heavy metal contaminated rice paddies^25^. In this study, two paddy soils contaminated with multiple heavy metals were studied in comparison to their unpolluted background soils. The purpose of present study was to characterize changes with metal pollution in abundances and community compositions of denitrifiers by molecular techniques (real-time PCR, denaturing gradient gel electrophoresis (DGGE), cloning and sequencing) with *nirK* and *nosZ* genes as the molecular markers, and to evaluate potential impacts by metal pollution on their N_2_O production and reduction rates of rice paddy soils.

## Results

### Soil properties and heavy metal pollution

As shown in Table 1, there were hardly differences in soil properties between polluted and background soils in a single site. Soil organic carbon (SOC) and total nitrogen (TN) was in a range of 20.40 g kg^−1^ to 28.77 g kg^−1^ and of 1.97 g kg^−1^ to 2.66 g kg^−1^ for the two soils, respectively. Both SOC and TN of the Yixing site were higher than that of the Dayu site. Soil pH of the Yixing (pH 6.16) was also higher than that of the Dayu site (pH 5.01), again without visible difference between polluted and background soil in a single site. As listed in Table 2, there were consistent differences in the total contents and available pools of Cd, Pb, Cu and Zn between polluted and background soils in a single site though the contents of a single heavy metal element varied by site. While the total Zn content in polluted soil was approximately 2 times higher from Dayu site than from Yixing site, the total Cd, Pb and Cu contents of polluted soils were similar between the two sites. Nemerow pollution index was estimated as 16.22 for Yixing and 24.95 for Dayu, clearly indicating a difference in pollution intensity between Dayu site and Yixing site.

### Soil potential denitrifying activity

**Figure 1:**
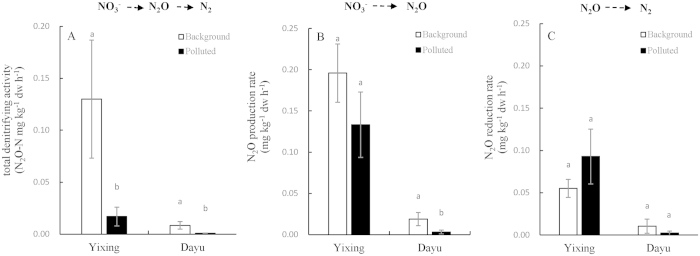
The total denitrifying activity (**A**), and the N_2_O production rate (**B**) and N_2_O reduction rate (**C**) in the background and polluted soils of Yixing and Dayu.

The changes in potential denitrifying activity with metal pollution of the two studied paddies is shown in Fig. 1, which varied between the two sites. Compared to the unpolluted background soils, the total denitrifying activity was significantly reduced by over 84% in polluted soils for both sites; The N_2_O producing rate (Fig. 1B) was seen slightly decreased but the N_2_O reducing rate unchanged (Fig. 1C) in the polluted paddies of both sites.

### Abundances of denitrifiers

As shown in Table 3, copy numbers of *nirK* gene of the two soils ranged from 2.86 × 10^8^ to 5.2 × 10^9^ g^−1^ dry soil, and were similar with those of *nosZ* genes, which ranged from 3.99 × 10^8^ to 4.29 × 10^9^ g^−1^ dry soil. Compared to background soil, copy numbers of *nirK* and *nosZ* gene were reduced in polluted soils by 47% and 39% at Yixing site, and by 73% and 48% at Dayu, respectively, corresponding to their soil metal pollution intensity. Accordingly, the ratio of *nirK* to *nosZ*, ranging from 0.8 to 1.2 across all samples, decreased in polluted soils over background soils at both sites.

### Community composition and phylogenetic analysis of denitrifiers

**Figure 2:**
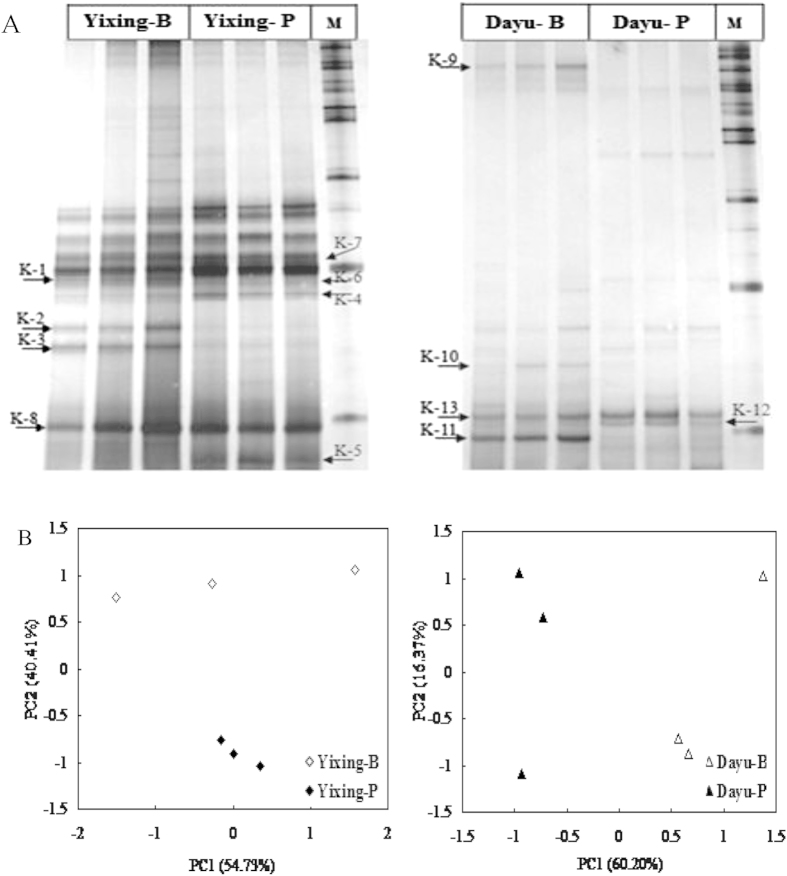
DGGE profiles (**A**) and principal component analysis (**B**) of *nirK* gene fragment from the background and polluted soils in Yixing and Dayu. M: 100 bp Marker. Arrows indicate the excised bands (K1–K13) for sequencing. Similar symbols in PCA plot indicate the triplicate samples.

**Figure 3:**
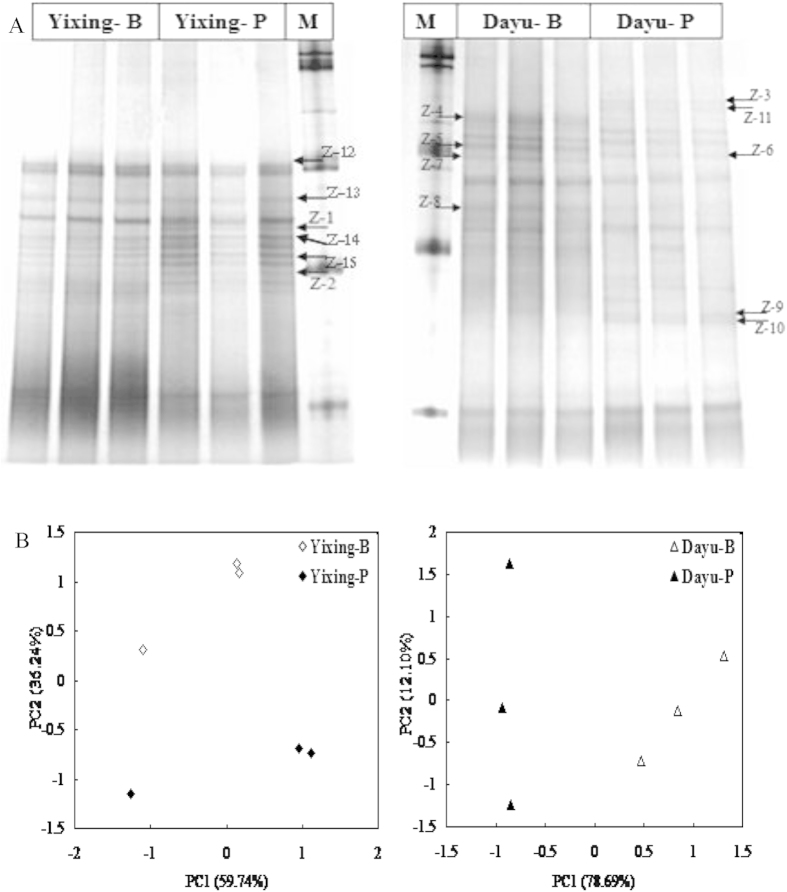
DGGE profiles (**A**) and principal component analysis (**B**) of *nosZ* gene fragment from the background and polluted soils in Yixing and Dayu. M: 100 bp Marker. Arrows indicate the excised bands (Z1–Z15) for sequencing. Similar symbols in PCA plot indicate the replicate samples.

The principal component analysis (PCA) was used to group sampled soils based on similarity in relative band intensity and position of DGGE profiles. PCA analysis of *nirK* and *nosZ* DGGE profiles at Yixing and Dayu sites yielded good summaries of data, as 95% and 76% for *nirK* and 96% and 90% for *nosZ* of the total variability was explained by the first two components (Figs 2 and 3), respectively. The *nirK* community profiles showed a well separation between polluted and background soils at each site, on PC2 at Yixing and on PC1 and PC2 at Dayu site (Fig. 2B). Compared to the corresponding background soils, the composition of *nosZ* community shifted significantly along PC2 at Yixing and along PC1 at Dayu site. Using redundancy analysis, we identified the factors which can best explained denitrifier community at the two sites (Fig. S1). In RDA bi-plot, axis1 and axis2 values explained 72% and 68% of the variability in *nirK* and *nosZ* profiles, respectively. Metals most important in explained *nirK* community structure were the Zn, Cd contents and Nemerow index, which were strongly related to the first axis while soil pH, TN and SOC were most important soil factors in determining *nosZ* community structure.

**Figure 4:**
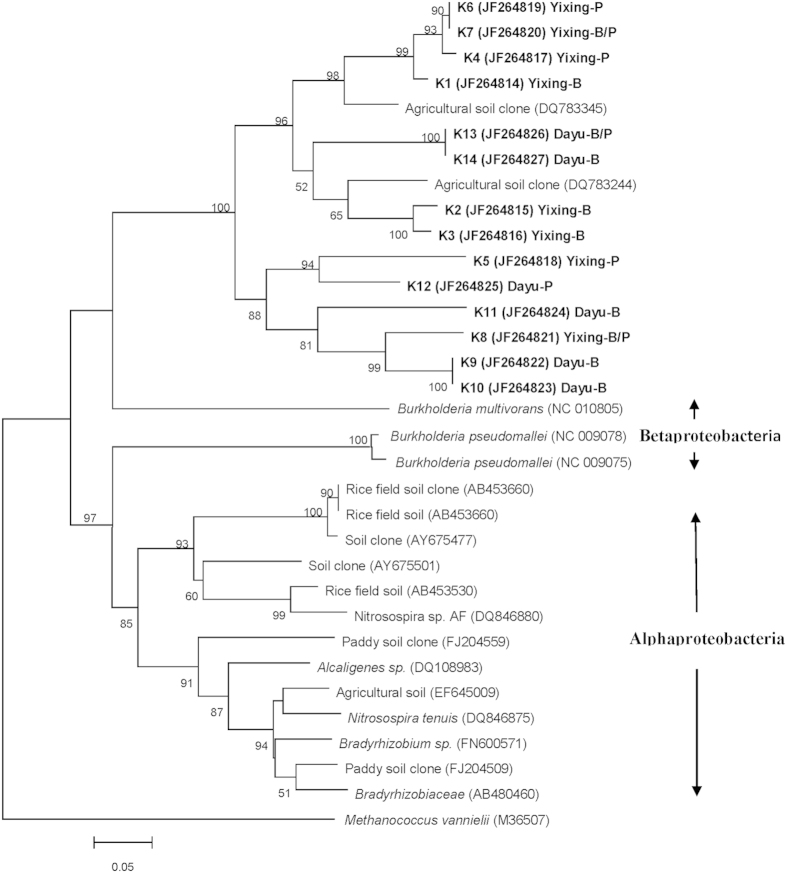
Neighbor-joining phylogenetic tree of *nirK* sequences retrieved from the numbered DGGE bands of Fig. 2A. Designation of the clones in bold includes the following information: excised DGGE band number, accession number in the parentheses, followed by the sampling plot the clone retrieved from. Bootstrap values (>50%) with 1000 replicates are indicated at branch points. Scale bar indicates 5 changes per 100 nucleotide positions.

**Figure 5:**
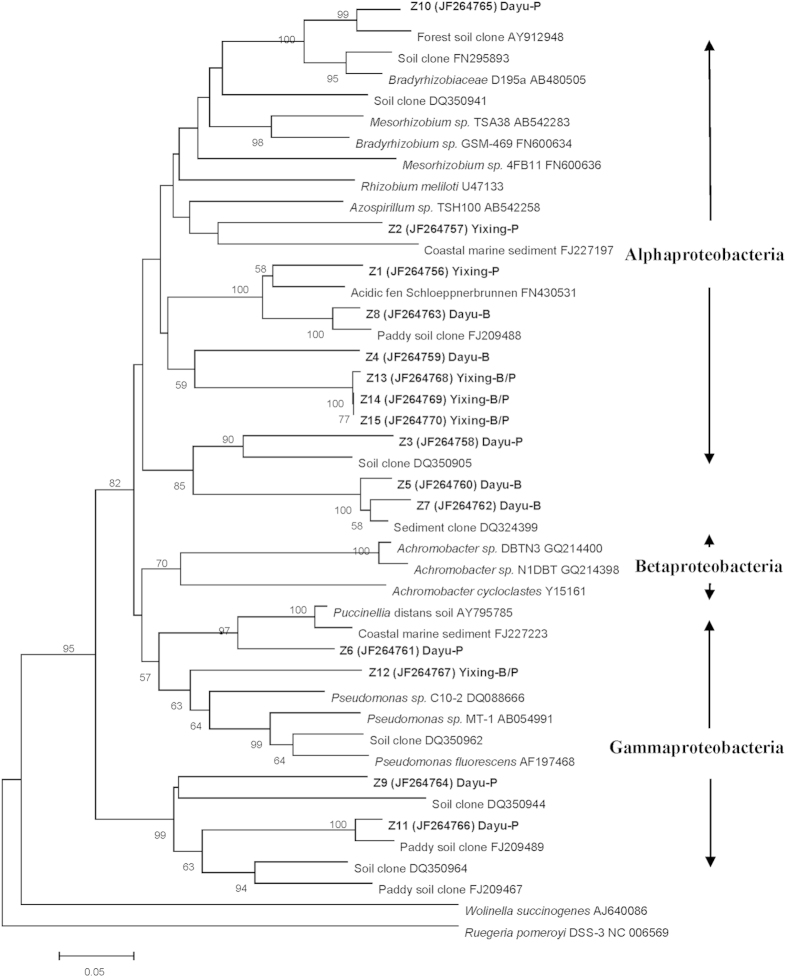
Neighbor-joining phylogenetic tree of *nosZ* sequences retrieved from the numbered DGGE bands of Fig. 3A. Designation of the clones in bold includes the following information: excised DGGE band number, accession number in the parentheses, followed by the sampling plot the clone retrieved from. Bootstrap values (>50%) with 1000 replicates are indicated at branch points. Scale bar indicates 5 changes per 100 nucleotide positions.

There were some similar DNA bands with different intensities in the DGGE profiles between polluted and background soils in a single site. Selected bands from the DGGE profiles were sequenced to identify the predominant taxa associated with these bands and to trace the change in specific taxa with metal pollution (Figs 4 and 5). In the phylogenetic tree of *nirK* constructed (Fig. 4), none of the sequences from the different soils were affiliated with neither α-*proteobacteria* nor β-*proteobacteria*. Considering the two sites as a whole, sequences from the background and polluted soils did not separate from each other clearly. However, in a single site, most of the clones in the polluted soils were grouped differently from those of the corresponding background soils. The phylogenetic analysis showed that most of the *nosZ* sequences belonged to α-proteobacteria, only one clone in the polluted soil of the Dayu site was grouped into γ-*proteobacteria*, whereas the other 2 clones of the Dayu soils was not belonging to the known clusters (Fig. 5). Therefore, most of the species from the polluted and background soils in a single site were separated from the other site.

## Discussion

Our results showed a strong impact of heavy metal pollution on denitrifiers in the two different locations. The gene abundances of both *nirK* (2.8 × 10^8^ ~ 5.2 × 10^9^) and *nosZ* (4.0 × 10^8^ ~ 4.3 × 10^9^) in the sampled soils were both within the ranges reported in literature. Dandie, *et al.*^26^ reported an average abundances of *nirK* of 1.2 × 10^9^ gene copies g^−1^ soil from agricultural zones of Thomas Brook Watershed, Canada. Chronakova, *et al.*^27^ gave a range of 9.1 × 10^7^ ~ 7.5 × 10^9^ copies g^−1^ soil of the *nosZ* gene copies in an upland pasture soil. The much lower copy numbers and activities of denitrifiers at Dayu than those at Yixing site, could be attributed to the lower soil pH of Dayu soil, since denitrification rate could be constrained decreased by acidic condition^28^, and denitrification enzymes activities had been commonly accepted as a sensitive indicator of acid soil reaction^29^,^30^.

Metal impact on denitrifier community abundance varied with elements. Reported by Magalhães, *et al.*^16^, copy numbers of both *nirK* and *nosZ* genes were decreased with exposure to Cu in 6-day lab incubation of an estuary sediment. However, such decreases could be recovered in prolonged incubation. For example, inhibition of N_2_O reduction with a mixture of Cd, Cu and Zn spiked in a sandy loam soil was recovered within two months of incubation^9^. In a study by Ruyters, *et al.*^17^, particularly, Zn-spiking (up to 5000 mg kg^−1^ dry soil) induced decrease in *nosZ* gene abundance, which was seen recoverable to the level value of the control in a grassland soil after 12 months incubation. In our case, the soils were contaminated by multiple metals at levels up to 400 mg kg^−1^ but with high Cd pollution intensity (12–25 folds). Sakadevan, *et al.*^5^ found a considerable inhibition of denitrification activity in a surface wetland sediment treated with multiple heavy metals, and they argued that Cd was the most inhibitory element followed by Zn and Cu among the studied metals. This could help to account for the consistent decrease in the abundances of *nirK* and *nosZ* in polluted soils from the two different sites but with commonly high Cd pollution intensity.

Metal pollution could lead to substantial changes in *nirK* or *nosZ* communities in soils. For example, metal spiking both at low and high doses resulted in a shift of composition of *nirK* communities in a silt clay soil after 18-month treatment^18^. As reported by Ruyters, *et al.*^17^, the intensity of dominant bands in the original community profile of *nosZ* community was decreased while other bands became dominant after spiked Zn treatment for one year, indicating the presence of some tolerant species to Zn spiking. However, in this study, DGGE technique provided no phylogenetic information for microbial community. As much, data here did not allow to identify which group responsible for the change in denitrifier in the polluted soil. Based on DGGE combined with amplicon sequencing, we found that composition of *nirK* and *nosZ* denitrifier changed to an extent under metal pollution in the polluted soils at both sites (Figs 2 and 3). Here, both the *nirK* and *nosZ* phylogenetic analysis showed that clones of the polluted and the background soils were grouped into different branches, suggesting presence of species tolerant to metals in the polluted soil. However, it was unclear whether the metal tolerant species in these heavy metal polluted soils were intrinsically tolerant to pollution or whether tolerance had been conferred by horizontal gene transfer (HGT). As reported by Jones, *et al.*^31^, the phylogenic diversity of denitrifiers had been raised by HGT of denitrification genes including *nirK, nirS* and *nosZ*. Here, some clones of polluted soils were widespread among different clusters, probably indicating the presence of different metal tolerant species in the heavy metal polluted soils. Prasad^32^ reported that the metal resistance capabilities were widespread among different bacterial genera. Particularly, several *Proteobacteria* could survive in highly metal-contaminated environments^33^,^34^. In a study^35^, metal tolerant species possess multiple heavy metal tolerance mechanism at the community level and metal-exposed microorganisms were tolerance to several metals but not only to one in an agricultural soil.

Holtan-Hartwig, *et al.*^9^ found a greater inhibition and thus a longer time required to recover, for N_2_O reduction than for N_2_O production, in a sandy loam spiked with metals regardless of a single or combination of them. Accordingly, they could conclude that heavy metal pollution could enhance N_2_O release from soil to the atmosphere. Whereas, De Brouwere, *et al.*^36^ reported a decline in prohibition by Zn on N_2_O reduction with prolonged incubation for up to one year while they did not detect any changes on N_2_O reduction activity in the field soils they sampled for lab experiment. In our study, both the total denitrifying activity and the N_2_O production rate were decreased under pollution in the two sites, but the N_2_O reduction rate was not affected by pollution (Fig. 1). Vásquez-Murrieta, *et al.*^37^ reported the production rate of N_2_O determined with the C_2_H_2_ inhibitor technique was significantly but negatively correlated with the concentrations of Pb, Cu and Zn in soils near a mine used for over 200 years in Mexico.

In addition to the effect of heavy metal pollution, denitrifier communities were also affected by soil properties. Soil inorganic N contents were decreased in polluted soils propably owing to inhibited decomposition rate^38^. Moreover, soil pH had been found to alter microbial composition directly by changes in functional microbial species^39^, and directly by changes in metal availablity^40^. Therefore, interactions of soil properties with metal pollution could alter the availability of metal in soils to microorganisms. However, there were different findings on the correlation of denitrification activity to abundance and/or community composition of denitrifiers among the studies. Henry, *et al.*^41^ and Miller, *et al.*^42^ reported a significantly increased denitrifying activity but a slight change in the denitrifier community with carbon additions. Ruyters, *et al.*^17^ reported different denitrifying activity but unchanged *nosZ* gene abundance between soils amended or not with hay. Attard, *et al.*^43^ reported a change in denitrifying activity after a shift in land uses, which was seen partly due to changes in denitrifier abundance but regardless of changes in the denitrifier community after a shift in land use. In this study, there were hardly difference in community structure of overall denitrifiers as there was no significant difference in copy number ratio of *nirK* to *nosZ* between the polluted and background soils at both sites (Table 3). This was, however, in disagreement with the change in N_2_O release with metal pollution. Yet, changes in N_2_O production and/or reduction activities under pollution were not consistent with that of *nirK* or *nosZ* abundances in a single site. Thus, the abundance of functional genes could be unlikely a predictor for the denitrifying activity in field soils with metal pollution, probably due to the presence of inactive microorganisms as well as to the presence of extracellular DNA in soil^44^. Alternatively, gene transcript numbers could be a better predictor since they reflect the active populations of the community. Therefore, more direct molecular measurements of shifts in microbial community gene expression patterns (for example, through environmental transcriptomics) is indeed highly desirable for future work.

In conclusion, as revealed with the principal component analysis of DGGE profiles and the phylogenetic analysis, community composition of both *nirK* and *nosZ* denitrifiers in rice paddies shifted to a certain extent under pollution from the two sites. The abundances of both *nirK* and *nosZ* genes were reduced significantly in the polluted soils. Being inconsistent with the changes in *nirK*/*nosZ* community composition or abundance, the total N_2_O denitrifying activity and the N_2_O production rate were reduced but the N_2_O reduction rate unchanged under pollution at both sites. Comparing to the N_2_O reduction, N_2_O production could be more sensitive to heavy metal pollution, which could potentially reduce the N_2_O release in the polluted rice paddies. This study indicates that metal pollution could potentially impact on soil N transformation process and thus on N_2_O emission from rice paddies. For heavy metal pollution had been already a critical issue for China’s sustainable agriculture, the potential impact of heavy metal pollution on soil nitrogen cycling would deserve an in-depth characterization, through environmental transcriptomics in the near future.

## Methods

### Site description

Two sites of rice fields with pollution were selected for this study. Site Yixing (31°24′N, 119°41′E, Yixing Municipality, Jiangsu) was located in a smelter area. Site Dayu (25°24′N, 114°18′E, Dayu County, Jiangxi) was situated in a zinc mining area. The polluted field at Yixing was polluted by waste discharge and atmospheric deposition from a metal smelter 0.5 km in distance down wind. Whereas, the polluted soil at Dayu site was polluted by irrigation with river water discharged from an upstream zinc mining ore. The smelter and mining activity had been taken place since late 1960’s in both sites. In each location, unpolluted rice paddies were selected in adjacent fields with same soil type but without distinct access to pollution by deposition or waste water irrigation. The soil at Yixing was derived from lacustrine deposit while the soil at Dayu from red earth on granite. Both sites were within the area controlled by a subtropical monsoon climate with a mean annual temperature ranging from 18 °C to 25 °C, and mean annual rainfall ranging from 1200 mm to 1450 mm. The rice paddies were cultivated normally with either rice-wheat rotation or double-cropping of rice in a year.

### Soil sampling

Soils were sampled before rice planting in spring 2009. Three composite samples were randomly collected from both polluted and background fields (0–15 cm), each of which contained 5 sub-samples collected in a “Z” shaped pattern with a distance of ~5 m from each other. The composite samples were mixed thoroughly and kept on ice until they were transported to the laboratory within two days after sampling. The gravel and visible plant detritus were removed, and then soil samples were sieved (<2 mm). One portion of sieved soil was stored at −20 °C for DNA extraction and another portion was stored at 4 °C for measuring denitrifying activity. The remaining soil was air-dried at room temperature before being analyzed for soil chemical and physical properties as described below.

### Measurements of soil properties and metal contents

Measurements of soil basic properties were conducted following the protocols described by Lu^45^. Briefly, soil pH was measured with a glass electrode using a 1/2.5 soil/water ratio. Soil organic carbon (SOC) was measured using wet digestion and oxidation with potassium dichromate. Total nitrogen (TN) was analyzed using the Kjeldahl method. Soil texture was determined with a hydrometer method after dispersion with 0.5 mol L^−1^ NaOH. Total heavy metal content was determined by digesting soil with a solution of HF/HClO_4_/HNO_3_ (10/2.5/2.5, v/v/v) followed by extraction with 1 M HCl. Cd content was determined with graphite furnace atomic absorption spectrometry (GFAAS, SpectrAA220Z, Varian, USA) while Pb, Cu and Zn contents were determined by flame atomic adsorption spectrophotometry (FAAS, TAS-986, China).

The Nemerow pollution index (P_n_)^46^ was used to evaluate the overall extent of heavy metal pollution and was calculated by the following equation:


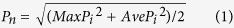


where, *P*_*i*_ is a single pollution intensity index of *i*th metal element with its measured concentration (*C*_*i*_) divided by the guideline standard of environmental quality (*R*S_i_), *MaxP*_*i*_ and *AveP*_*i*_ is the maximum and average pollution intensity of the analyzed metals in a given soil, respectively.

### DNA extraction and real time PCR assay

Total DNA was extracted from 0.25 g of fresh soil by the PowerSoil™ DNA Isolation Kit (Mo Bio Laboratories Inc., CA) according to the manufacturer’s protocol. The primers and thermal cycling procedures for real-time PCR are listed in Table 4. Each reaction was performed in a 25 μl volume containing 15 ng DNA, 1 μl 10 μM of each primer and 12.5 μl SYBR premix EX Taq TM (Takara Shuzo, Shiga, Japan). Melting curve analysis of PCR products was conducted following each assay to confirm that the fluorescence signal originated from the specific PCR products but not from primer-dimers or other artifacts. PCR products were checked for the correct size by comparing standardized molecular weight ladder by electrophoresis on 1.5% agarose gel. A plasmid standard containing the target region was generated for each primer set using total DNA extracted from soil. The amplified PCR products of *nirK* and *nosZ* genes were purified using PCR solution purification kit (Takara Shuzo, Shiga. Japan), ligated into pEASY-T3 cloning vector (Promega, Madison,WI) and cloned into *Escherichia coli* DH5α. Clones containing correct inserts were chosen as standards for real-time PCR. Standard curves were generated using triplicate 10-fold dilutions of plasmid DNA. High amplification efficiencies of *nirK* (102%) and *nosZ* (96%) were obtained for gene quantification, with R^2^ values being 0.995 and 0.991, respectively.

### PCR-DGGE of *nirK* and *nosZ* containing community

Total extracted DNA from each soil sample was amplified with the nirK876-GC and nirK1040 primers^47^, and with the nosZ2F-GC and nosZ2R primers^48^ for the *nirK* and *nosZ* genes, respectively (Table 4). The GC clamp described by Muyzer, *et al.*^49^ was added to 5′ end of primer. PCR reaction was performed in an Eppendorf autothermer Cycler (Bio-Rad) using 25 μl reaction volume. The DNA concentration of each sample was adjusted to 10 ng μl^−1^ and used as template for PCR amplification. The reaction mixture contained 12.5 μl Go Taq® Green Master Mix (Promega, Madison,WI), 1 μl of 10 μM of each primer, and 10 ng DNA template. For DGGE analysis, PCR products were separated on 8% (w/v) polyacrylamide gels (acrylamide-bisacrylamide [37.5:1]) with a 49% to 62% denaturing gradients for *nirK* and a 45% to 70% for *nosZ* using the D-Code universal mutation detection system (Bio-Rad Laboratories, Hercules, CA, USA), respectively. Denaturant was defined that containing 8% acrylamide with 7 M urea and 40% deionized formamide. Electrophoresis ran 5 min at 200 V at first, and then 500 min at 140 V at a temperature of 60 °C in a DGGE chamber containing approximately of 1 × TAE buffer. Gels were silver stained and scanned using a gel document system (Bio-Rad, USA).

### Sequencing and phylogenetic analysis

Dominant bands from DGGE gels were detected and numbered (K1-K13 and Z1-Z15) on the basis of their relative intensity or specific positions across all treatments. The numbered bands of DGGE gels were excised, transferred to clean Eppendorf tubes and smashed to release the DNA in to 25 μl of sterile water at 4 °C. The eluted DNA was reamplified as templates using primer sets described above but without GC clamp, purified and ligated with the p-EASY T3 cloning kits (Promega, Madison, WI) according to the protocol. The positive colonies were amplified again using the above primers with GC clamp and checked by DGGE. The correct one was finally selected for sequencing. The retrieved sequences were compared with GenBank data base sequences using BLAST (Basic Local Alignment Search Tool) (http://www.ncbi.nlm.nih/gov/blast/) to search for best matches. The sequences of DGGE bands have been deposited in GenBank under the accession numbers JF264814-JF264826 (*nirK*) and JF264756-JF264766 (*nosZ*). Phylogenetic analysis was performed using MEGA version 4.0 and the neighbor-joining trees were constructed using the p-distance model with bootstrap value of 1,000.

### Measurement of potential denitrifying activity

The total denitrifying activity was determined by the acetylene (C_2_H_2_) inhibition method described by Tiedje, *et al.*^50^. 20 g moist soil at 60% WHC (water holding capacity) was placed in 250 mL glass bottles, and treated with 20 mL of a substrate solution containing 1 mM glucose and 1 mM KNO_3_. Each soil sample was divided into two subsamples to be incubated with or without C_2_H_2_ (10% v/v). N_2_O evolved from the bottles was measured using a gas chromatograph (Agilent 7890D, Santa Clara, CA, USA) equipped with an electron capture detector (ECD). A mixture of argon and methane (5%) was used as the carrier gas. The oven temperature was controlled at 55 °C, and the temperature of the ECD was set at 330 °C. Concentration of N_2_O was quantified by comparing the peak area with those of reference gas (Nanjing special gas factory). The linear rates of N_2_O production over time were observed within 6 h after initiating the incubations. In the treatment with C_2_H_2_, the net rate of N_2_O production was calculated as the N_2_O production rate. In the treatment without C_2_H_2_, the net rate of N_2_O production was calculated as the total denitrifying activity. The difference between the N_2_O production rate and total denitrifying activity was treated as the N_2_O reduction rate.

### Data processing and statistical analysis

The data presentation and treatment was conducted using Microsoft Excel 2013. Results were expressed as means with one standard deviation. Digital DGGE images were analyzed with Quantity One image analysis software (Version 4.0, Bio-Rad, USA). This software identifies the bands occupying the same position in the different lanes of the gel and also measures the intensity of the identified bands. All statistical analyses were performed using the SPSS 16.0 for Windows. Principal component analysis (PCA) and redundancy analysis (RDA) of the DGGE profiles were conducted using the Canoco 4.5 software.

## Additional Information

**How to cite this article**: Liu, Y. *et al.* Abundance, composition and activity of denitrifier communities in metal polluted paddy soils. *Sci. Rep.* **6**, 19086; doi: 10.1038/srep19086 (2016).

## Supplementary Material

Supplementary Information

## Figures and Tables

**Table 1:** Soil physicochemical properties of the studied soil samples.

Site	Plot	SOC (g kg^−1^)	TN (g kg^−1^)	pH (H_2_O)
Yixing	Background	28.77 ± 1.11	2.69 ± 0.08	6.16 ± 0.05
	Pollution	25.27 + 0.53	2.22 ± 0.05	6.08 ± 0.05
Dayu	Background	20.40 ± 0.82	2.06 ± 0.07	5.20 ± 0.05
	Pollution	22.30 ± 1.32	1.97 ± 0.13	5.01 ± 0.05

**Table 2:** Total and available heavy metal contents (mg kg^−1^) and Nemerow pollution index (Means ± S.D.) of the soils studied.

Sample	Total content (mg kg^−1^)				Available pool (mg kg^−1^)				Nemerow index
	Cd	Pb	Cu	Zn	Cd	Pb	Cu	Zn	
Yixing-B	0.45 ± 0.01b	59.79 ± 13.28b	42.19 ± 0.77b	104.89 ± 3.92b	0.21 ± 0.03b	12.04 ± 1.31b	7.23 ± 0.79b	16.86 ± 1.69b	1.20 ± 0.04b
Yixing-P	6.60 ± 0.27a	354.52 ± 87.34a	82.56 ± 1.90a	172.92 ± 2.67a	4.65 ± 0.33a	70.90 ± 2.27a	24.88 ± 0.22a	20.81 ± 3.38a	16.22 ± 0.94a
Dayu-B	0.36 ± 0.19b	68.89 ± 7.10b	36.01 ± 0.17b	117.14 ± 5.58b	0.05 ± 0.00b	13.69 ± 0.93b	6.49 ± 0.75b	3.75 ± 0.33b	1.20 ± 0.32b
Dayu-P	9.60 ± 2.07a	329.76 ± 10.60a	92.28 ± 4.58a	368.45 ± 16.71a	6.96 ± 0.38a	85.78 ± 2.67a	33.57 ± 3.1a	120.63 ± 6.76a	24.95 ± 6.22a

Different lowercase characters indicate significant difference (*p* < 0.05) between polluted and background soils in a single site.

**Table 3:** Denitrifier (*nirK* and *nosZ*) gene abundance (copy numbers g^−1^ dry soil) and the relative ratios (Means ± S.D.) of the soils studied.

Sample	*nirK* (×10^8^)	*nosZ* (×10^8^)	Ratio of *nirK* to *nosZ*
Yixing-B	52.03 ± 4.79a	42.88 ± 2.94a	1.22 ± 0.14a
Yixing-P	27.70 ± 4.37b	26.00 ± 4.12b	1.07 ± 0.01a
Dayu-B	10.50 ± 1.90a	7.77 ± 0.77a	1.22 ± 0.13a
Dayu-P	2.86 ± 0.38b	3.99 ± 1.31b	0.81 ± 0.43a

Different lowercase characters indicate significant difference (*p* < 0.05) between polluted and background soils in a single site.

**Table 4:** Primer sets and thermal profiles used for qPCR (*) and DGGE (^#^) of the functional target genes.

Target gene	Primer set	Sequence (5′ to 3′)	Size	Thermal cycling profile	Reference
*nirK*	nirK876* nirK876-GC^#^	ATYGGCGGVCAYGGCGA	165 bp	95 °C (5 min); 40 cycles of 95 °C (1 min), 58 °C (1 min), and 72 °C (1 min).*	Henry *et al.*^47^
	nirK1040	GCCTCGATCAGRTTRTGGTT		94 °C (10 min); 30 cycles of 94 °C (1 min), 58 °C (1 min), and 72 °C (1 min).^#^	
*nosZ*	nosZ2F * nosZ2F-GC ^#^	CGCRACGGCAASAAGGTSMSSGT	265 bp	95 °C (3 min); 40 cycles of 95 °C (1 min), 58 °C (1 min), and 72 °C (1 min).*	Henry *et al.*^48^
	nosZ2R	CAKRTGCAKSGCRTGGCAGAA		94 °C (10 min); 30 cycles of 94 °C (1 min), 56 °C (1 min), and 72 °C (1 min).^#^	

The GC clamp (5′-CCGCCGCGCGGCGGGCGGGGCGGGGGCACGGGG-3′) was attached to the 5′ end of the primer^49^.
