# Supplementary material for: Abundance, composition and activity of denitrifier communities in metal polluted paddy soils
Source: Sci Rep. 2016 Jan 7;6:19086. doi: 10.1038/srep19086 (PMC4703955; doi:10.1038/srep19086)

Supplementary Information (SI)

Abundance, composition and activity of denitrifier communities in metal polluted paddy soils

Yuan Liu[1,2], Yongzhuo Liu[1,3],, Huimin Zhou[1], Lianqing Li[1], Jinwei Zheng[1], Xuhui Zhang[1], Jufeng Zheng[1], Genxing Pan[1]*

[1]Institute of Resource, Ecosystem and Environment of Agriculture, Nanjing Agricultural University, 1 Weigang, Nanjing 210095, China.

[2] Department of Bioengineering, College of Life Sciences, Huaibei Normal University, 235000, Huaibei, Anhui Province, China

[3] College of Resource and Environment Sciences, Henan Institute of Science and Technology, Xinxiang City, Henan 453003, China

*** Corresponding author**: Genxing Pan

Address: Institute of Resource, Ecosystem and Environment of Agriculture, Nanjing Agric. University, 1 Weigang, Nanjing 210095, China

Tel/Fax: +86 25 8439 6027

†**Email:** pangenxing@aliyun.com; genxingpan@njau.edu.cn

Supplementary Figure

Fig. S1 Redundancy analysis ordination plots of relations between DGGE patterns of *nirK* (A) and *nosZ* (B) gene and soil properties from the soil samples at the two sites. Soil properties included Nemerow toxicity index, soil pH, SOC, TN, total concentration of Cd, Pb, Cu and Zn. Yixing-B and Yixing-P, background and polluted soil from site Yixing; Dayu-B and Dayu-P, background and polluted soil from site Dayu.

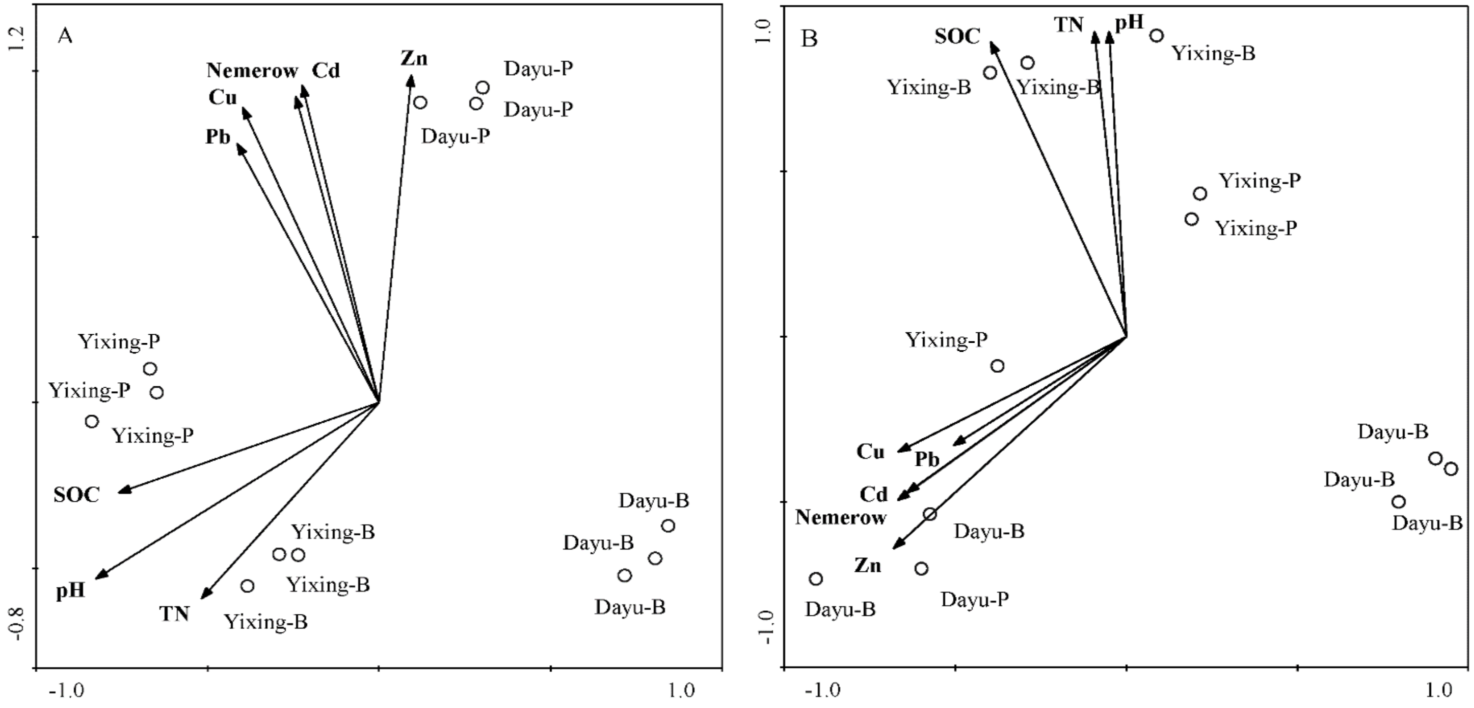

Supplement: Supplementary Information [file srep19086-s1.pdf]
